# *Klebsiella pneumoniae* is associated with increased mortality versus *Escherichia coli* in carbapenem-susceptible *Enterobacterales* bacteraemia: a Peruvian cohort

**DOI:** 10.1093/jacamr/dlag164

**Published:** 2026-08-10

**Authors:** Cesar Copaja-Corzo, Carlos Mazza-Diaz, Carlos Tairo-Cerron, Roxana Sandoval-Ahumada, José Ballena-López, Javier Flores-Cohaila, Brayan Miranda-Chavez, Giancarlo Pérez-Lazo

**Affiliations:** Unidad de Investigación para la Generación y Síntesis de Evidencias en Salud, Universidad San Ignacio de Loyola, Lima, Perú; Servicio de Infectología, Hospital Nacional Edgardo Rebagliati Martins, EsSalud, Lima, Perú; Servicio de Infectología, Hospital Nacional Guillermo Almenara Irigoyen, EsSalud, Lima, Perú; Servicio de Infectología, Hospital Nacional Guillermo Almenara Irigoyen, EsSalud, Lima, Perú; Departamento de Patología Clínica, Hospital Nacional Guillermo Almenara Irigoyen, EsSalud, Lima, Perú; Servicio de Infectología, Hospital Nacional Guillermo Almenara Irigoyen, EsSalud, Lima, Perú; Escuela de Postgrado, Universidad Continental, Huancayo, Perú; Centro de Estudios e Investigación en Educación Médica y Bioética, EDUCAB-UPT, Facultad de Ciencias de la Salud, Universidad Privada de Tacna, Tacna, Perú; Servicio de Infectología, Hospital Nacional Guillermo Almenara Irigoyen, EsSalud, Lima, Perú

## Abstract

**Objectives:**

To identify predictors of all-cause 30-day mortality in bloodstream infections (BSI) caused by carbapenem-susceptible *Escherichia coli* and *Klebsiella pneumoniae*, with emphasis on bacterial species and the extended-spectrum β-lactamase (ESBL) phenotype.

**Methods:**

This was a single-centre retrospective cohort study of adults with a first episode of BSI due to carbapenem-susceptible *E. coli* or *K. pneumoniae* at a referral hospital in Lima, Peru (June 2020–December 2023). Primary outcome: All-cause 30-day mortality. A multivariable Cox model was built, with covariate selection guided by a directed acyclic graph, and the species × ESBL interaction was tested.

**Results:**

Of the 403 patients (median age 66 years, 55.8% men; 58.6% ESBL+), 83 (20.6%) died. Mortality was higher in patients with *K. pneumoniae* than in those with *E. coli* (26.8% versus 17.4%; log-rank *P* = 0.018). In the multivariable analysis, *K. pneumoniae* was an independent predictor [adjusted HR (aHR) 1.72; 95% CI, 1.09–2.73; *P* = 0.021], as was a Charlson score ≥6 (aHR 8.43; 95% CI, 3.33–21.3; *P* < 0.001). ESBL was not an independent predictor (aHR 1.45; 95% CI, 0.92–2.29; *P* = 0.113), nor was the species × ESBL interaction (*P* = 0.43). The effect of *K. pneumoniae* was consistent across all sensitivity analyses (aHR 1.66–1.94).

**Conclusions:**

*K. pneumoniae* nearly doubled mortality compared with *E. coli*, independently of ESBL, which did not predict mortality in this cohort of carbapenem-susceptible isolates. These findings challenge the assumption of prognostic homogeneity within carbapenem-susceptible *Enterobacterales* and support species stratification.

## Introduction

Carbapenem-susceptible *Escherichia coli* and *Klebsiella pneumoniae* are the most common causes of bloodstream infections (BSI) due to *Enterobacterales*. Together, both species account for 70%–80% of these BSI.^1,2^ Despite the availability of effective therapy, 30-day mortality reaches 10%–20%.^3–5^ This burden is frequently underestimated because research in the field has been directed towards carbapenem-resistant strains.

Comparative studies of carbapenem-resistant versus carbapenem-susceptible strains have consistently used the susceptible group as a low-risk reference, reporting its mortality solely as a comparator without analysing the factors that determine it.^3,6–10^ In this way, susceptible strains, which constitute the majority group, are treated as a homogeneous group with a favourable prognosis, an assumption that has not been formally evaluated within this group for specific species and resistance phenotypes. However, the determinants of BSI mortality in this group remain uninvestigated.

In particular, it has not been assessed whether variables such as bacterial species or the extended-spectrum β-lactamase (ESBL) phenotype independently predict mortality in this population. Prior work on both has focused on resistant strains.^11,12^ From Latin America, the available evidence is even more limited: only one study evaluated the effect of ESBL on mortality in *Enterobacterales* BSI in a cohort of 85 patients without stratifying by carbapenem susceptibility.^13^ The available evidence has not addressed these predictors in susceptible strains as a primary objective, which precludes stratification of mortality risk within this group. Accordingly, we conducted a retrospective cohort study to identify predictors of 30-day mortality in BSI caused by carbapenem-susceptible *E. coli* and *K. pneumoniae*, with an emphasis on bacterial species and the ESBL phenotype, in a hospital in Lima, Peru.

## Methods

### Ethics

This study was approved by the Institutional Research Ethics Committee of HNGAI (code 102-2024). The research was conducted in accordance with the principles of the Declaration of Helsinki. Informed consent was not sought because of the retrospective design of the study and the use of de-identified data. The data were securely stored and safeguarded by the research team.

### Study design and setting

We conducted a retrospective cohort study at the Hospital Nacional Guillermo Almenara Irigoyen (HNGAI), a referral centre with 1116 inpatient beds in Lima, Peru. HNGAI is part of the Social Health Insurance system (EsSalud). Patients with BSI caused by carbapenem-susceptible strains of *E. coli* or *K. pneumoniae* [carbapenem-susceptible *Enterobacterales* (CSE)] were included. The study period was from 1 June 2020 to 31 December 2023. The findings are reported according to the STROBE (Strengthening the Reporting of Observational Studies in Epidemiology) guidelines (Supplementary Material S1, available as Supplementary data at *JAC-AMR* Online).

### Participants and data collection

We included adult patients (≥18 years) admitted to hospital with at least one blood culture positive for carbapenem-susceptible *E. coli* or *K. pneumoniae*, defined as simultaneous susceptibility to ertapenem, meropenem, and imipenem according to the Clinical and Laboratory Standards Institute (CLSI) breakpoints; in addition, patients had to present a clinical picture suggestive of infection (fever, hypothermia, hypotension, chills, or leucocytosis/leucopenia).^14^ Only the first BSI episode in each patient was considered. Patients were excluded if they met any of the following criteria: (i) polymicrobial BSI, (ii) insufficient data to determine the outcome or model covariates, (iii) not admitted to the hospital, (iv) transferred to centres outside the EsSalud network before completing follow-up, or (v) carbapenem resistance.

**Figure 1.**
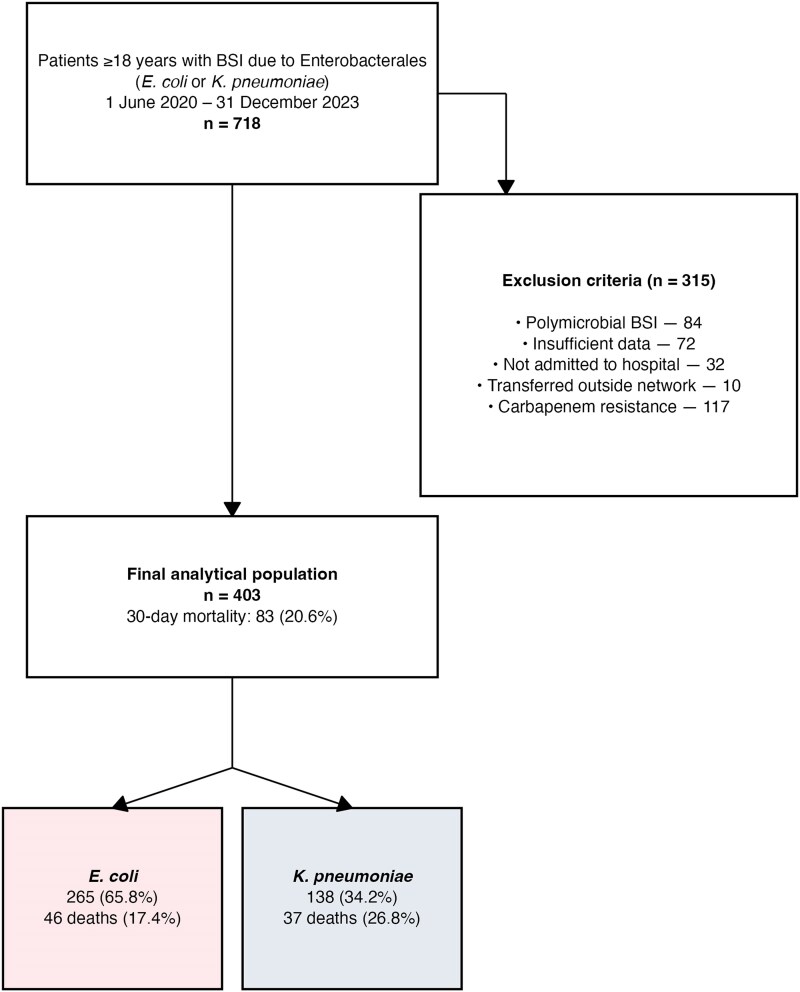
Patient selection.

Clinical and microbiological data were extracted from electronic health records by two infectious diseases physicians using a standardized data collection form in REDCap, with the cohort split between the two extractors. Demographic variables, clinical characteristics of the episode, antimicrobial treatment data, susceptibility profiles, and outcomes were recorded. Ambiguous records were discussed and resolved by consensus between the extractors, with a review of the electronic health records when necessary. The number and percentage of missing data per variable are detailed in Table S1. The main-model covariates had no missing data; the only variable with more than 5% missingness was the time to effective therapy (TTE; 7.9%), which is descriptive. Patients with missing data in essential variables (age, sex, species, susceptibility, or outcome) were excluded during preprocessing, as shown in the flowchart (Figure 1).

### Variables and definitions

The primary outcome was all-cause 30-day mortality. The index date (day 0) was defined as the date of blood culture collection for each patient. Follow-up was extended to 30 days or until death, whichever occurred first. Vital status was verified through the EsSalud electronic health record system, which integrates inpatient, outpatient, and mortality records at the national level.

The exposure variable evaluated was the bacterial species (*E. coli* or *K. pneumoniae*), with *E. coli* as the reference category. The ESBL phenotype was assessed as a secondary exposure. Model covariates were selected *a priori* using a directed acyclic graph (DAG; Figure S1). The Charlson Comorbidity Index (CCI)^15^ was categorized as 0–2, 3–5, and ≥6. The following descriptive variables were additionally recorded: age, sex, Sequential Organ Failure Assessment (SOFA) score, Pitt Bacteraemia Score, septic shock at onset, admission to the intensive care unit (ICU), hospitalization in the previous 90 days, appropriateness of empirical antimicrobial therapy, TTE, and length of hospital stay. The TTE was defined as the interval in hours between blood culture collection (day 0) and the first antimicrobial dose active against the isolate, according to the final susceptibility report. Empirical therapy was defined as all antimicrobials administered between blood culture collection and release of the final susceptibility report and was classified as discordant when no agent with *in vitro* activity against the isolate was administered during that window.

### Microbiological methods

Blood cultures were processed at the HNGAI microbiology laboratory following standardized procedures. Bacterial identification and antimicrobial susceptibility testing were performed using the automated VITEK-2 GN system (bioMérieux, Marcy l’Étoile, France) with the AST-N403 card, which includes automated detection of the ESBL phenotype (category agreement 97.7%; very major error 2.7%; major error 1.9%, per the manufacturer’s validation). No independent phenotypic (e.g. double-disk synergy) or genotypic confirmation of ESBL production was performed beyond VITEK-2 automated detection. Minimum inhibitory concentration (MIC) values were interpreted according to the CLSI breakpoints for each study year. Carbapenem susceptibility was defined as simultaneous susceptibility to ertapenem, meropenem, and imipenem.

### Statistical analysis

All analyses were performed using R version 4.4.2. Statistical significance was set at *P* < 0.05. Categorical variables were compared using the chi-square test or Fisher’s exact test, as appropriate, and continuous variables were compared using the Mann–Whitney U test. The primary analysis used complete cases; no imputation was performed.

A multivariable Cox regression model was constructed to estimate the effect of bacterial species on 30-day mortality. Covariates were selected *a priori* using a DAG (Figure S1): species, ESBL phenotype, CCI (0–2, 3–5, ≥6), and BSI source. Severity markers (SOFA and septic shock) and the adequacy of empirical therapy were treated as mediators—not confounders—and excluded from the main model; these variables are presented in Table 1 and were incorporated into the Cox model in the sensitivity analyses (Table S2). The proportional hazards assumption was assessed for all covariates using Schoenfeld residuals, multicollinearity was assessed using the generalized variance inflation factor (GVIF), and linearity was assessed using Martingale residuals. No adjustment for multiple comparisons was applied to the exploratory species × ESBL analyses; these results should be interpreted as hypothesis generating.

**Table 1.** Baseline characteristics by species (N = 403)

Characteristic	Overall (N = 403)	*E. coli* (N = 265)	*K. pneumoniae* (N = 138)	*P*
Age, years	66.0 (52.0–76.0)	67.0 (56.0–75.0)	64.0 (47.0–76.0)	0.215
Men, *n* (%)	225 (55.8%)	139 (52.5%)	86 (62.3%)	0.074
CCI	5.0 (3.0–6.0)	5.0 (3.0–6.0)	4.0 (3.0–6.0)	0.205
CCI 0–2	86 (21.3%)	52 (19.6%)	34 (24.6%)	0.456
CCI 3–5	177 (43.9%)	117 (44.2%)	60 (43.5%)	
CCI ≥6	140 (34.7%)	96 (36.2%)	44 (31.9%)	
Baseline SOFA score	4.0 (2.0–6.0)	4.0 (2.0–6.0)	4.0 (3.0–6.0)	0.180
Pitt Bacteraemia Score	2.0 (0.0–3.0)	2.0 (0.0–3.0)	2.0 (0.0–3.0)	0.067
Pitt ≥4	76 (18.9%)	44 (16.6%)	32 (23.2%)	0.142
Septic shock at onset	105 (26.1%)	66 (24.9%)	39 (28.3%)	0.543
ICU admission, day 1	56 (13.9%)	27 (10.2%)	29 (21.2%)	0.004
ICU admission (any time)	72 (17.9%)	35 (13.2%)	37 (26.8%)	0.001
Prior hospitalization within 90 days	214 (53.1%)	142 (53.6%)	72 (52.2%)	0.870
Acquisition mode				0.001
Community-acquired	70 (17.4%)	58 (21.9%)	12 (8.7%)	
Healthcare-associated	130 (32.3%)	87 (32.8%)	43 (31.2%)	
Nosocomial	203 (50.4%)	120 (45.3%)	83 (60.1%)	
ESBL + phenotype	236 (58.6%)	151 (57.0%)	85 (61.6%)	0.432
Source: Urinary	144 (35.7%)	110 (41.5%)	34 (24.6%)	<0.001
Source: Intra-abdominal	114 (28.3%)	89 (33.6%)	25 (18.1%)	
Source: Other	145 (36.0%)	66 (24.9%)	79 (57.2%)	
Adequate source control	119/215 (55.3%)	69/139 (49.6%)	50/76 (65.8%)	0.033
Appropriate empirical therapy	317 (79.3%)	212 (80.3%)	105 (77.2%)	0.553
TTE, h	2.0 (1.0–20.0)	2.0 (1.0–17.0)	2.0 (1.0–20.0)	0.260
TTE ≤24 h	287 (77.4%)	198 (78.0%)	89 (76.1%)	0.788
TTE >24 h	84 (22.6%)	56 (22.0%)	28 (23.9%)	
30-day mortality	83 (20.6%)	46 (17.4%)	37 (26.8%)	0.036
Length of hospital stay, days	24.0 (14.0–40.0)	22.0 (13.0–36.0)	28.5 (15.0–48.0)	0.015

Medians (IQR) or *n* (%). *P*: Mann–Whitney U or chi-square/Fisher’s exact test, as appropriate. For 30-day mortality, the *P*-value shown (0.036) corresponds to the chi-squared test on crude proportions. The log-rank test on time-to-event, reported in the synopsis and main text, yielded *P* = 0.018. Percentages were calculated using non-missing denominators (*n* = 402 for ICU admission day 1; *n* = 400 for appropriate empirical therapy; *n* = 371 for time to effective therapy; *n* = 215 for adequate source control). Missing data: ICU admission day 1 (1), appropriate empirical therapy (3), time to effective therapy (32). CCI, Charlson Comorbidity Index; ESBL, extended-spectrum β-lactamase; ICU, intensive care unit; SOFA, Sequential Organ Failure Assessment; TTE, time to effective therapy.

As a secondary analysis, patients were classified into four groups according to species and ESBL phenotype. Kaplan–Meier curves were constructed using log-rank tests, and the species × ESBL interaction was evaluated using a likelihood ratio test and species-stratified models. These analyses were considered exploratory.

## Results

A total of 403 patients with CSE BSI were included in the study period (1 June 2020 to 31 December 2023; Figure 1). *E. coli* was the most frequent microorganism, with 265 cases (65.8%), followed by *K. pneumoniae* with 138 (34.2%). The median age was 66 years (IQR, 52–76), 225 patients (55.8%) were men, the median Charlson score was 5 (IQR, 3–6), the median SOFA score was 4 (IQR, 2–6), 105 patients (26.1%) presented with septic shock at onset, and 236 (58.6%) had the ESBL phenotype. Appropriate empirical therapy was administered to 317 (79.3%) patients. Acquisition mode differed between species, with a higher proportion of nosocomial infection in *K. pneumoniae* and of community-acquired infection in *E. coli* (*P* = 0.001). Among the 215 patients in whom source control was applicable, adequate source control was achieved more frequently in *K. pneumoniae* than in *E. coli* (65.8% versus 49.6%; *P* = 0.033). The baseline characteristics of the species are presented in Table 1.

**Figure 2.**
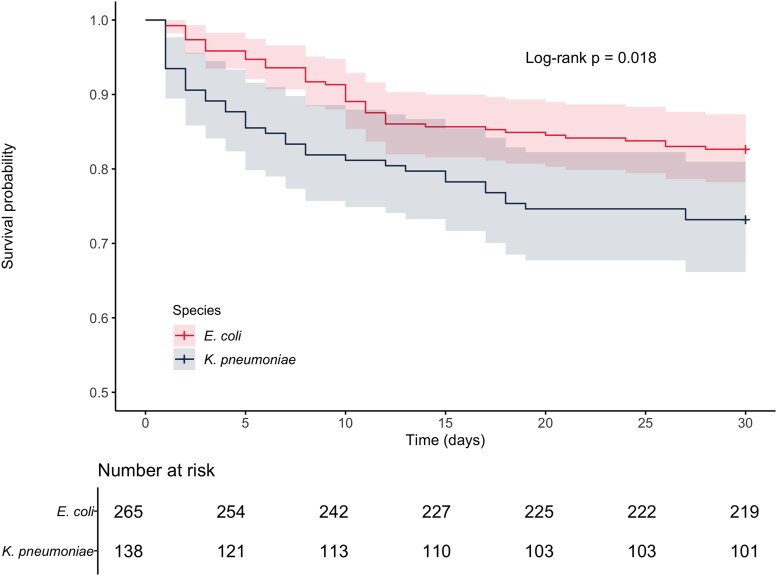
Kaplan–Meier curves for 30-day survival by species (*E. coli* versus *K. pneumoniae*).

Thirty-day mortality was 20.6% (83/403) in the overall cohort and was higher in *K. pneumoniae* than in *E. coli* (37/138, 26.8% versus 46/265, 17.4%; log-rank *P* = 0.018). The estimated 30-day survival was 82.6% (95% CI, 78.2%–87.3%) for *E. coli* and 73.2% (95% CI, 66.2%–81.0%) for *K. pneumoniae* (Figure 2).

In the multivariable Cox model, *K. pneumoniae* [adjusted HR (aHR) 1.72; 95% CI, 1.09–2.73; *P* = 0.021] and a CCI ≥6 (aHR 8.43; 95% CI, 3.33–21.3; *P* < 0.001) were the independent predictors of 30-day mortality. The ESBL phenotype was not an independent predictor after adjustment (aHR 1.45; 95% CI, 0.92–2.29; *P* = 0.113); BSI source was likewise not significantly associated, although the ‘Other’ category—predominant in *K. pneumoniae*—showed a non-significant trend towards higher mortality (aHR 1.48; 95% CI, 0.87–2.50; *P* = 0.147; Table 2). Access to active therapy was comparable between species: both the appropriateness of empirical therapy (*E. coli* 80.3% versus *K. pneumoniae* 77.2%; *P* = 0.553) and empiric carbapenem use—the active option against ESBL-producing strains—(47.5% versus 42.0%; *P* = 0.342; Table S3) were similar; moreover, appropriateness of empirical therapy was not associated with mortality in the univariable analysis (HR 0.75; 95% CI, 0.46–1.23; *P* = 0.254). The effect of *K. pneumoniae* remained robust across all sensitivity analyses (aHR 1.66–1.94; Table S2) and in a model stratified by acquisition mode (aHR 1.76; 95% CI, 1.11–2.80; *P* = 0.017).

**Table 2.** Predictors of 30-day mortality (*N* = 403, 83 events)

Characteristic	N	Univariable HR	95% CI	*P*	Multivariable aHR	95% CI	*P*
*Species (ref:* E. coli*)*	403						
*K. pneumoniae*		1.68	1.09–2.59	0.019	1.72	1.09–2.73	0.021
ESBL+ (ref: ESBL−)	403	1.35	0.86–2.11	0.197	1.45	0.92–2.29	0.113
CCI (ref: 0–2)	403						
3–5		2.95	1.14–7.62	0.026	3.33	1.28–8.65	0.013
≥6		7.03	2.80–17.6	<0.001	8.43	3.33–21.3	<0.001
BSI source (ref: Urinary)	403						
Intra-abdominal		0.93	0.52–1.66	0.805	1.13	0.63–2.03	0.674
Other		1.33	0.81–2.20	0.257	1.48	0.87–2.50	0.147
*Baseline SOFA score*	403	1.34	1.27–1.42	<0.001	—	—	—
*Septic shock*	403	3.32	2.16–5.11	<0.001	—	—	—
*Appropriate empirical therapy*	400	0.75	0.46–1.23	0.254	—	—	—

Multivariable model: species, ESBL, CCI (categorized) and BSI source. C-statistic = 0.708 (95% CI, 0.651–0.766). No multicollinearity was detected (maximum GVIF 1.07). Proportional hazards assumption: global Schoenfeld test *P* = 0.021; species-specific *P* = 0.102.

**Figure 3.**
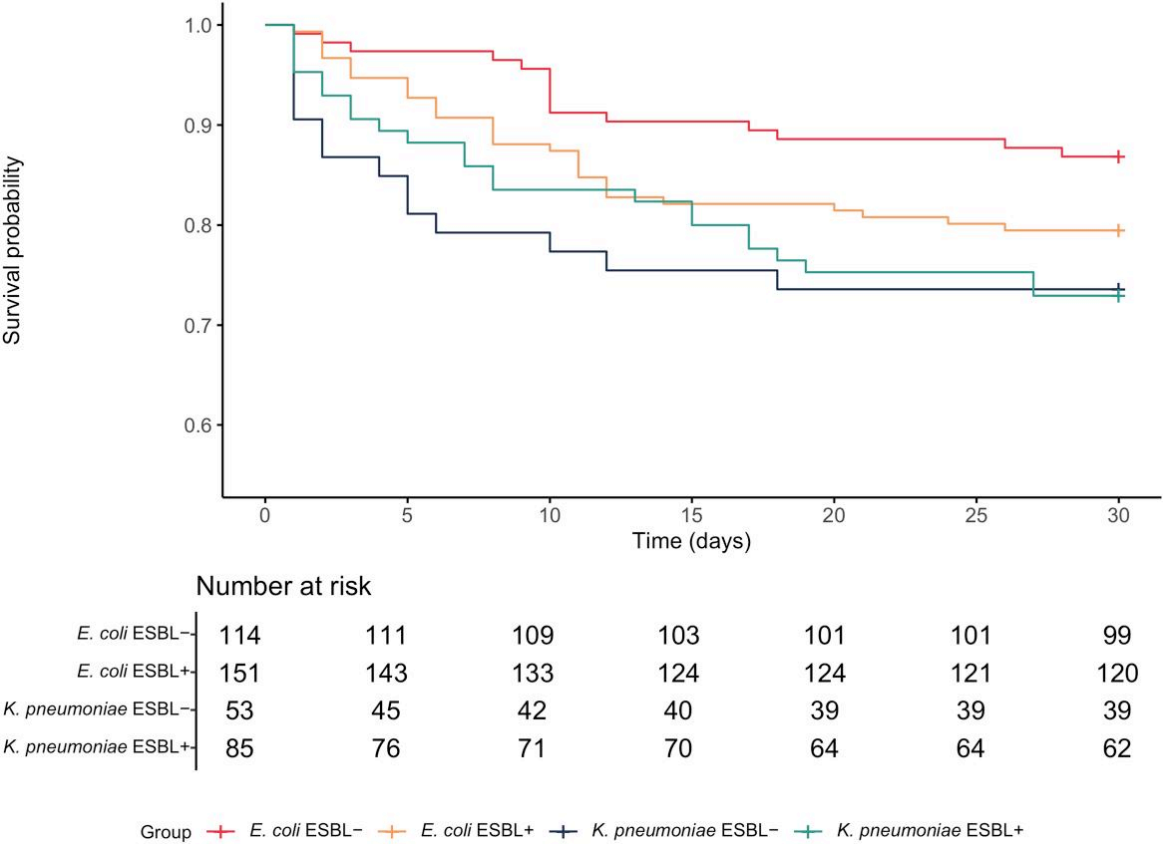
Kaplan–Meier curves for 30-day survival by species × ESBL phenotype subgroup.

When the four species × ESBL subgroups were analysed, both *K. pneumoniae* subgroups showed higher mortality than ESBL-negative *E. coli*: ESBL-negative *K. pneumoniae* (aHR 2.18; 95% CI, 1.04–4.53; *P* = 0.038) and ESBL-positive *K. pneumoniae* (aHR 2.57; 95% CI, 1.33–4.98; *P* = 0.005). ESBL-positive *E. coli* did not differ significantly from the reference (aHR 1.72; 95% CI, 0.91–3.24; *P* = 0.093; Table 3, Figure 3). No interaction between species and ESBL was observed (likelihood ratio test *P* = 0.43). In species-stratified models (Table S2, Panel C), the ESBL phenotype showed a borderline association with mortality in *E. coli* (aHR 1.87; 95% CI, 0.98–3.57; *P* = 0.059), which did not reach conventional significance, and was not observed in *K. pneumoniae* (aHR 1.21; 95% CI, 0.62–2.37; *P* = 0.570).

**Table 3.** Cox model by species × ESBL group (*N* = 403, 83 events)

Group	aHR	95% CI	*P*
*E. coli ESBL*− (Reference)			
*E. coli* ESBL+	1.72	0.91–3.24	0.093
*K. pneumoniae* ESBL−	2.18	1.04–4.53	0.038
*K. pneumoniae* ESBL+	2.57	1.33–4.98	0.005
CCI 0–2 (Reference)			
CCI 3–5	3.28	1.26–8.53	0.015
CCI ≥6	8.25	3.26–20.9	<0.001
Source: Urinary (Reference)			
Source: Intra-abdominal	1.16	0.64–2.08	0.630
Source: Other	1.53	0.90–2.62	0.119

Adjusted for CCI (categorized) and BSI source, both selected by DAG. C-statistic = 0.705 (95% CI, 0.648–0.761).

## Discussion

### Main finding

In this cohort of 403 patients with CSE BSI, *K. pneumoniae* was independently associated with higher 30-day mortality than *E. coli* (aHR 1.72), an effect that remained robust across all sensitivity analyses, including models that additionally conditioned on severity mediators (aHR up to 1.94). Overall mortality was 20.6%, nearly twice the 10.6% reported by the prospective European EURECA cohort,^3^ a difference that probably reflects the greater severity of our population (median SOFA score, 4; 26.1% with septic shock). Comorbidity burden was the strongest predictor of 30-day mortality (CCI ≥6: aHR 8.43), exceeding the effect of species. This finding reinforces that host factors dominate prognosis in CSE BSI, with species as a secondary but clinically relevant stratifier.

### Bacterial species as a predictor of mortality

This finding is consistent with the multinational INCREMENT study (909 patients, 12 countries), in which ESBL-producing *K. pneumoniae* showed a 30-day mortality of 33.7% versus 17.4% for ESBL-producing *E. coli*.^11^ Our study extends this observation by showing that *K. pneumoniae* maintained higher mortality with and without the ESBL phenotype, without evidence of interaction, indicating that the excess risk is attributable to species and not to the resistance mechanism. In contrast, Kuo *et al.* found no difference in mortality after propensity score matching in 1097 patients with community-onset BSI in Taiwan,^16^ a discrepancy that may be explained by the exclusively community-onset setting and the lower baseline severity of their cohort. In a related line of evidence on inter-species virulence heterogeneity, Fostervold *et al.* showed, in 1078 *Klebsiella* BSI in Norway, that *K. variicola* had a 30-day mortality of 16.9% versus 10.1% in non-MDR *K. pneumoniae* (aHR 1.86),^17^ suggesting that species-level differences in virulence are not unique to the *Klebsiella* versus *Escherichia* comparison. Taken together, these data underline that resistance and virulence are not synonymous: a resistant isolate is harder to treat but not necessarily more lethal. One possible reason for the increased mortality linked to *K. pneumoniae* could be the presence of virulence factors that enhance its ability to cause invasive infections. These include a prominent polysaccharide capsule that aids in evading phagocytosis and complement-mediated killing,^18^ iron acquisition through siderophores, and biofilm formation on medical devices or damaged tissues. Such mechanisms might lead to a greater bacterial load, spread beyond the urinary tract, delayed source control, or more intense systemic inflammation. This is consistent with our findings, in which non-urinary origins—particularly intra-abdominal and soft-tissue sites—were more frequent in *K. pneumoniae* cases than in *E. coli* cases. Nonetheless, this remains a hypothesis, as our centre lacked molecular characterization of the isolates (capsular type, sequence type, siderophore genes, and hypervirulence markers).

### The ESBL phenotype does not predict mortality in CSE

The ESBL phenotype was not an independent predictor of mortality in this cohort. A recent meta-analysis of 22 studies (4607 patients with BSI due to ESBL-producing *Enterobacterales*) showed that predictors of mortality are host and clinical severity factors—neutropenia, septic shock, Pitt score ≥4—, not ESBL production *per se*.^19^ A similar pattern has been observed in a related meta-analysis of predominantly non-BSI infections,^20^ in which the association between ESBL and mortality lost statistical significance after multivariable adjustment, although the extrapolation to BSI should be cautious. The most widely accepted explanation is that the excess mortality attributed to ESBL arises from the delay in receiving effective therapy^21^; in patients with CSE, for whom carbapenems provide an effective empirical option, this delay is minimized. In contrast, Adrianzén *et al.* identified ESBL as a predictor of mortality in 85 patients with BSI in Lima in 2013.^13^ Notably, in that study, death occurred in 63.3% of patients with ESBL BSI, a figure that suggests a context in which timely access to carbapenems may have been more limited.

### Implications and future research

CSE is not a homogeneous low-risk group. Our findings suggest that the identification of *K. pneumoniae* in blood cultures, available before the antibiogram, may prompt earlier clinical awareness of the higher mortality risk in these infections, pending prospective validation. Surveillance systems that pool CSE as a single block could stratify outcomes by species, particularly in regions with a high prevalence of *K. pneumoniae*. Although this is, to our knowledge, the first study in Latin America to evaluate predictors of mortality in patients within CSE as a primary objective, multicentre studies incorporating genomic characterization are needed to confirm the species-level differences and identify the responsible virulence determinants. Future studies should evaluate whether different resistance mechanisms (ESBL, KPC, OXA-48) have a differential impact on mortality or whether, as our data for ESBL suggest, antimicrobial resistance does not translate into greater virulence regardless of the mechanism involved.

### Limitations

This study had some limitations. Its retrospective, single-centre design limits generalisability and entails the absence of some potentially relevant clinical and microbiological variables. Our centre does not have molecular methods, so we could not type the ESBL (e.g. CTX-M or other ESBLs), confirm the coexistence of AmpC, distinguish species within the *K. pneumoniae* complex (such as *K. variicola*), or detect hypervirulent variants (hvKp); this precludes confirming that the difference between species is explained by intrinsic virulence or resistance factors. The quantitative MIC for cephalosporins was likewise not recorded. The study period (2020–23) partially overlapped with the COVID-19 pandemic, which may have influenced baseline severity. Finally, although the main model had adequate power for the species effect (83 events, EPV 13.8), it was underpowered to detect interactions or small subgroups: the four-group species × ESBL analysis had an overall EPV of 11.9, which fell below 10 in the species-stratified models (Table S2, Panel C); these results should therefore be interpreted as exploratory.

In conclusion, in BSI due to CSE, *K. pneumoniae* had nearly double the mortality compared with *E. coli* (aHR 1.66–1.94), regardless of the ESBL phenotype. Carbapenem susceptibility does not equate to a uniformly favourable prognosis, and species identification—available before the antibiogram—may warrant consideration as a stratification variable in future prospective studies of mortality risk in these infections.

## Supplementary Material

dlag164_Supplementary_Data

## Data Availability

The de-identified dataset underlying this article is available upon reasonable request to the corresponding author.

## Supplementary data

Figure S1 and Tables S1–S3 are available as Supplementary data at *JAC-AMR* Online.
