# Supplementary material for: Klebsiella pneumoniae is associated with increased mortality versus Escherichia coli in carbapenem-susceptible Enterobacterales bacteraemia: a Peruvian cohort
Source: JAC Antimicrob Resist. 2026 Aug 10;8(4):dlag164. doi: 10.1093/jacamr/dlag164 (PMC13453535; doi:10.1093/jacamr/dlag164)
Supplement: Supplementary file 1 [file dlag164_supplementary_data.docx]

**Supplementary Material**

*Klebsiella pneumoniae doubles the mortality of Escherichia coli in bloodstream infections due to carbapenem-susceptible Enterobacterales: a cohort study from Peru*

**Contents**

**Supplementary Figure S1** — Directed acyclic graph (DAG) of the assumed causal structure

**Supplementary Material 1** — STROBE Statement checklist

**Supplementary Table S1** — Missing data per variable

**Supplementary Table S2** — Sensitivity analyses

**Supplementary Table S3** — Empirical antibiotic therapy by family and species

**Supplementary Figure S1. Directed acyclic graph (DAG) of the assumed causal structure.**

Roles are defined relative to the primary exposure (bacterial species): SOFA and septic shock mediate the effect of species on mortality; empirical adequacy is a treatment-process variable on the pathway from the resistance phenotype (ESBL) to mortality, not a confounder of the species effect. The Charlson Comorbidity Index and BSI source are the confounders retained in the main model.

**Supplementary Material 1 — STROBE Statement**

**Checklist of items that should be included in reports of cohort studies**

**Manuscript:** *Klebsiella pneumoniae* doubles the mortality of *Escherichia coli* in bloodstream infections due to carbapenem-susceptible Enterobacterales: a cohort study from Peru

| **#** | **Item** | **Recommendation** | **Reported in manuscript** | **Page no.** | **Notes** |
| --- | --- | --- | --- | --- | --- |
| **Title and Abstract** |  |  |  |  |  |
| 1 | (a) Indicate the study design with a commonly used term in the title or the abstract | Retrospective cohort | Title; Abstract (Methods) | p. 1–2 | ✅ |
| 1 | (b) Provide in the abstract an informative and balanced summary of what was done and what was found | Objective, methods, key results, conclusions | Abstract (Objective, Methods, Results, Conclusions) | p. 2 | ✅ |
| **Introduction** |  |  |  |  |  |
| 2 | Background / rationale | Scientific background and rationale for the investigation | Introduction ¶1–2 | p. 3 | ✅ |
| 3 | Objectives | Specific objectives, including any pre-specified hypotheses | Introduction ¶3 | p. 3 | ✅ |
| **Methods** |  |  |  |  |  |
| 4 | Study design | Present key elements of study design early in the paper | Methods — Study design and setting | p. 3 | ✅ |
| 5 | Setting | Describe the setting, locations, and relevant dates, including periods of recruitment and follow-up | Methods — Study design (HNGAI, 1 June 2020 – 31 December 2023) | p. 3 | ✅ |
| 6 | (a) Eligibility criteria | Give the eligibility criteria, and the sources and methods of selection of participants. Describe methods of follow-up | Methods — Participants and data collection | p. 4 | ✅ |
| 6 | (b) Sources and methods of selection | Consecutive blood cultures; first BSI episode per patient | Methods — Participants and data collection | p. 4 | ✅ |
| 7 | Variables | Clearly define all outcomes, exposures, predictors, potential confounders, and effect modifiers | Methods — Variables and definitions | p. 4 | ✅ |
| 8 | Data sources / measurement | For each variable of interest, give sources of data and details of methods of assessment | Methods — Participants and data collection; Microbiological methods | p. 4–5 | ✅ |
| 9 | Bias | Describe any efforts to address potential sources of bias | Methods — DAG-guided confounder selection; consensus resolution of ambiguous records during data extraction | p. 5 | ✅ |
| 10 | Study size | Explain how the study size was arrived at | Methods — Statistical analysis (census of consecutive episodes; no a priori sample size calculation) | p. 5 | ✅ No a priori sample size calculation; convenience cohort |
| 11 | Quantitative variables | Explain how quantitative variables were handled in the analyses | Methods — Statistical analysis (CCI categorised owing to non-linearity in Martingale residuals) | p. 5 | ✅ |
| 12 | (a) Statistical methods | Describe all statistical methods, including those used to control for confounding | Methods — Statistical analysis | p. 5 | ✅ |
| 12 | (b) Subgroups and interactions | Describe any methods used to examine subgroups and interactions | Methods — Statistical analysis (four-group analysis; species × ESBL interaction by likelihood ratio test) | p. 5 | ✅ |
| 12 | (c) Missing data | Explain how missing data were addressed | Methods — Statistical analysis (complete-case analysis; 3/403 missing for main-model covariates) | p. 5 | ✅ |
| 12 | (d) Loss to follow-up | If applicable, explain how loss to follow-up was addressed | Methods (vital status via EsSalud national EHR; fixed 30-day follow-up) | p. 4 | ✅ No loss to follow-up |
| 12 | (e) Sensitivity analyses | Describe any sensitivity analyses | Methods — Statistical analysis (+SOFA+shock; outlier exclusion) | p. 5 | ✅ |
| **Results** |  |  |  |  |  |
| 13 | (a) Participants | Report numbers of individuals at each stage of the study | Figure 1 (flowchart: 718 → 315 excluded → 403) | p. 6 | ✅ |
| 13 | (b) Reasons for non-participation | Give reasons for non-participation at each stage | Figure 1 (5 exclusion criteria with n) | p. 6 | ✅ |
| 13 | (c) Flow diagram | Consider use of a flow diagram | Figure 1 | p. 6 | ✅ |
| 14 | (a) Descriptive data | Give characteristics of study participants | Table 1; Results ¶1 | p. 6 | ✅ |
| 14 | (b) Missing data | Indicate number of participants with missing data for each variable of interest | Table 1 footnote (ICU admission day 1: 1; appropriate empirical therapy: 3; TTE: 32) | p. 6 | ✅ |
| 14 | (c) Follow-up time | Summarise follow-up time (median, total amount) | Results (fixed 30-day follow-up; administrative censoring) | p. 6 | ✅ |
| 15 | Outcome data | Report numbers of outcome events or summary measures over time | Results ¶2 (83/403, 20.6%) | p. 6 | ✅ |
| 16 | (a) Unadjusted and adjusted estimates | Give unadjusted estimates and, if applicable, confounder-adjusted estimates with 95% CIs | Table 2 (univariable HR + multivariable aHR) | p. 7 | ✅ |
| 16 | (b) Category boundaries | Report category boundaries when continuous variables were categorised | Methods — Variables (CCI 0–2, 3–5, ≥6) | p. 4 | ✅ |
| 16 | (c) Relative and absolute risk | If relevant, consider translating estimates of relative risk into absolute risk | Results ¶3–4 (absolute mortality by group; KM probabilities) | p. 6–7 | ✅ |
| 17 | Other analyses | Report other analyses done — e.g. analyses of subgroups and interactions, and sensitivity analyses | Results ¶4; Table 3; Supplementary Table S1 | p. 7–8 | ✅ |
| **Discussion** |  |  |  |  |  |
| 18 | Key results | Summarise key results with reference to study objectives | Discussion — Main finding | p. 8 | ✅ |
| 19 | Limitations | Discuss limitations, taking into account sources of potential bias or imprecision | Discussion — Limitations | p. 9 | ✅ |
| 20 | Interpretation | Give a cautious overall interpretation considering objectives, limitations, multiplicity, and other relevant evidence | Discussion — Bacterial species as a predictor of mortality; The ESBL phenotype does not predict mortality in CSE; Implications and future research | p. 8–10 | ✅ |
| 21 | Generalisability | Discuss the generalisability (external validity) of the study results | Discussion — Limitations (single-centre, tertiary referral) | p. 9 | ✅ |
| **Other Information** |  |  |  |  |  |
| 22 | Funding | Give the source of funding and the role of the funders for the present study | Funding | p. 11 | ✅ |

**Abbreviations**

aHR, adjusted hazard ratio; BSI, bloodstream infection; CCI, Charlson Comorbidity Index; CI, confidence interval; CSE, carbapenem-susceptible Enterobacterales; DAG, directed acyclic graph; EHR, electronic health record; ESBL, extended-spectrum β-lactamase; HNGAI, Hospital Nacional Guillermo Almenara Irigoyen; HR, hazard ratio; ICU, intensive care unit; KM, Kaplan–Meier; SOFA, Sequential Organ Failure Assessment; STROBE, Strengthening the Reporting of Observational Studies in Epidemiology; TTE, time to effective therapy.

STROBE checklist adapted from: von Elm E, et al. Strengthening the Reporting of Observational Studies in Epidemiology (STROBE) statement: guidelines for reporting observational studies. Ann Intern Med. 2007;147(8):573-577. Available at https://www.strobe-statement.org

**Supplementary Table S1. Missing data per variable (N = 403).**

| **Variable** | **n missing** | **%** |
| --- | --- | --- |
| **Main model covariates** |  |  |
| Species, ESBL, Charlson Comorbidity Index, BSI source, 30-day mortality, time to event | 0 | 0.0 |
| **Descriptive / sensitivity variables** |  |  |
| Age, sex, Pitt score, SOFA, septic shock, length of stay, ICU admission (any), prior hospitalization | 0 | 0.0 |
| ICU admission on day 1 | 1 | 0.2 |
| Appropriateness of empirical therapy | 3 | 0.7 |
| Time to effective therapy (TTE) | 32 | 7.9 |

Missing data computed on the analytic cohort (N = 403). Complete-case analysis was used for all models; no imputation was performed. ESBL, extended-spectrum β-lactamase; ICU, intensive care unit; SOFA, Sequential Organ Failure Assessment; TTE, time to effective therapy.

**Supplementary Table S2. Sensitivity analyses**

**Manuscript:** *Klebsiella pneumoniae* doubles the mortality of *Escherichia coli* in bloodstream infections due to carbapenem-susceptible Enterobacterales: a cohort study from Peru

**Panel A — Sensitivity analyses**

| **Analysis** | **N** | **Events** | **aHR (*K. pneumoniae*)** | **95% CI** | **p** |
| --- | --- | --- | --- | --- | --- |
| Main model (CCI + Source + ESBL) | 403 | 83 | 1.72 | 1.09–2.73 | 0.021 |
| + SOFA + shock + appropriate empirical therapy | 400 | 83 | 1.94 | 1.22–3.09 | 0.005 |
| + SOFA + shock (without appropriateness) | 403 | 83 | 1.91 | 1.20–3.04 | 0.006 |
| Main model without outlier (\|deviance residual\| > 3) | 402 | 82 | 1.66 | 1.04–2.64 | 0.033 |
| Main model stratified by acquisition mode (Friedman 2002) | 403 | 83 | 1.76 | 1.11–2.80 | 0.017 |

**Panel B — Species × ESBL interaction**

| **Analysis** | **N** | **Events** | **aHR** | **95% CI** | **p** |
| --- | --- | --- | --- | --- | --- |
| Effect of ESBL in *E. coli* | 265 | 46 | 1.72 | 0.91–3.24 | — |
| Effect of ESBL in *K. pneumoniae* | 138 | 37 | 1.18 | 0.61–2.30 | — |
| HR ratio (interaction) | — | — | 0.69 | 0.27–1.73 | 0.43 |

**Panel C — Species-stratified analysis**

| **Species** | **N** | **Events** | **aHR (ESBL+ vs ESBL−)** | **95% CI** | **p** |
| --- | --- | --- | --- | --- | --- |
| *E. coli* | 265 | 46 | 1.87 | 0.98–3.57 | 0.059 |
| *K. pneumoniae* | 138 | 37 | 1.21 | 0.62–2.37 | 0.570 |

**Main model:** species, ESBL, CCI (categorised), BSI source; covariates selected a priori by means of a directed acyclic graph (Supplementary Figure S1). CCI categorised owing to non-linearity in Martingale residuals.

**Panel A, severity-adjusted model:** SOFA and septic shock are partial mediators in the DAG; their inclusion blocks part of the causal pathway (C-statistic = 0.838). Missing data: 3/403 (0.7%).

**Panel A, outlier exclusion:** the excluded observation corresponded to a case with extreme Martingale residuals (|deviance| > 3); exclusion did not change the direction or significance of the main effect.

**Panel B:** interaction assessed by likelihood ratio test (joint model with interaction term). Simple effects reported from the joint model differ slightly from the stratum-specific models in Panel C because of a different baseline hazard.

**Panel C:** separate Cox models fitted within each species (*E. coli* and *K. pneumoniae*) to estimate the effect of ESBL phenotype on 30-day mortality, adjusted for CCI and BSI source. Events per variable (EPV) < 10 in both strata; these results should be interpreted as exploratory.

aHR, adjusted hazard ratio; BSI, bloodstream infection; CCI, Charlson Comorbidity Index; CI, confidence interval; DAG, directed acyclic graph; ESBL, extended-spectrum β-lactamase; EPV, events per variable; SOFA, Sequential Organ Failure Assessment.

**Supplementary Table S3. Empirical antibiotic therapy by family and species (N = 403).**

| **Antibiotic family** | **Overall (N = 403)** | ***E. coli (N = 265)*** | ***K. pneumoniae (N = 138)*** | **p** |
| --- | --- | --- | --- | --- |
| Carbapenems | 184 (45.7%) | 126 (47.5%) | 58 (42.0%) | 0.342 |
| Third-generation cephalosporins | 67 (16.6%) | 51 (19.2%) | 16 (11.6%) | 0.069 |
| Fourth-generation cephalosporins | 5 (1.2%) | 3 (1.1%) | 2 (1.4%) | 1.000* |
| Quinolones | 12 (3.0%) | 10 (3.8%) | 2 (1.4%) | 0.233* |
| Aminoglycosides | 2 (0.5%) | 1 (0.4%) | 1 (0.7%) | 1.000* |
| Colistin | 5 (1.2%) | 2 (0.8%) | 3 (2.2%) | 0.344* |
| Tigecycline | 2 (0.5%) | 0 (0.0%) | 2 (1.4%) | 0.117* |
| Piperacillin/tazobactam | 125 (31.0%) | 72 (27.2%) | 53 (38.4%) | 0.028 |
| Other | 14 (3.5%) | 9 (3.4%) | 5 (3.6%) | 1.000* |

Values are n (%). Percentages were calculated using species-specific denominators. Categories are not mutually exclusive (patients could receive more than one empirical agent). p-values were obtained using the chi-square test with Yates correction or Fisher’s exact test (*) when expected cell counts were <5.
